# Residual pulmonary vascular obstruction computed with ventilation/perfusion single photon emission computed tomography/computed tomography to predict the risk of venous thromboembolism recurrence in patients with pulmonary embolism: protocol for a cohort study (PRONOSPECT)

**DOI:** 10.1016/j.rpth.2025.102867

**Published:** 2025-04-22

**Authors:** Pierre–Yves Le Roux, Philippe Robin, Romain Le Pennec, Cécile Tromeur, Francis Couturaud, Julien Asselineau, Grégoire Le Gal, Pierre–Yves Salaun

**Affiliations:** 1 Médecine nucléaire, Université de Brest, Centre Hospitalier Universitaire de Brest (CHU Brest), Groupe d’Études Tumeurs et Biothérapies Oncologiques (GETBO), Unité Mixte de Recherche (UMR) 1304, Brest, France; 2 Pneumologie, Université de Brest, CHU Brest, Pneumologie, GETBO, UMR 1304, Brest, France; 3 Direction de la recherche clinique et de l’innovation, Université de Brest, CHU Brest, Brest, France; 4 Department of Medicine, Ottawa Hospital Research Institute, Ottawa, Ontario, Canada

**Keywords:** lung scintigraphy, pulmonary embolism, recurrence, residual pulmonary vascular obstruction, ventilation/perfusion SPECT/CT

## Abstract

**Background:**

In patients with pulmonary embolism (PE), identifying predictors of recurrence is important to risk-stratify patients and tailor anticoagulation duration. After PE, a significant proportion of patients demonstrate residual pulmonary vascular obstruction (RPVO) on lung imaging. However, the exact prognostic significance of RPVO for venous thromboembolism recurrence remains unclear.

**Objectives:**

The primary objective is to assess whether RPVO on ventilation/perfusion (V/Q) single photon emission computed tomography (SPECT)/CT imaging after completion of 3 to 6 months of anticoagulation is an independent predictor of venous thromboembolism recurrence in patients with PE.

**Methods:**

The PRONOSPECT trial is a prospective multicenter cohort study. Participants are patients who experienced an objectively proven PE, provoked by a minor transient risk factor or unprovoked; who have been treated with anticoagulant therapy for 3 to 6 uninterrupted months; and for whom anticoagulation will not be prolonged. A standardized baseline patient assessment will be conducted including V/Q SPECT/CT imaging, collection of other potential predictor variables, and a functional evaluation. Anticoagulants will be withdrawn at the 3- or 6-month points from diagnosis and patients will be followed up for up to 2 years.

**Conclusion:**

The PRONOSPECT cohort study has the potential to determine whether the presence of RPVO on V/Q SPECT/CT imaging predicts the risk of recurrence in patients with PE in whom there remains a doubt on duration of anticoagulation.

## Essentials

• After pulmonary embolism (PE), predicting recurrence helps guide treatment duration.
• Residual pulmonary vascular obstruction on lung scan (SPECT/CT) may predict recurrence.
• This multicenter cohort study follows patients with PE after 3-6 months of anticoagulation.
• Findings could help predict the risk of recurrence in patients with PE.

## 1 Introduction

### 1.1 Background and rationale

#### 1.1.1 Current approach to decide the duration of anticoagulation therapy in patients with pulmonary embolism

One of the main complications of pulmonary embolism (PE) is recurrence, which can be fatal in approximately 10% of cases [1]. Anticoagulant therapy is very effective in preventing recurrence, but this needs to be balanced against the risk of major bleeding. Current clinical practice guidelines recommend at least 3 to 6 months of oral anticoagulation therapy (OAT) after a first episode of venous thromboembolism (VTE) [2]. However, the benefit of OAT is not maintained after discontinuation of treatment [3]. Accordingly, the choice is between OAT of short and limited duration (3-6 months), and indefinite treatment.

Patients with PE can be stratified in 3 groups according to the risk of recurrence. Patients with a first episode of PE, which was provoked by a major transient risk factor (eg, major surgery), have a very low risk of recurrence after OAT discontinuation (<2% at 1 year); consequently, 3 months of OAT is recommended. In contrast, when PE occurred in patients with persistent major risk factors (eg, cancer) or in the absence of any transient (major or minor) risk factor, the risk of recurrence is high (>9% at 1 year) [4,5] and indefinite OAT is recommended [6]. Between these 2 phenotypes, the risk of recurrence is intermediate (4%-7%). This group includes PE provoked by a weak transient risk factors (eg, leg injury associated with reduced mobility and short admission to hospital with an acute illness). If prolonged anticoagulation is currently not recommended in this population [2,6], identifying patient subgroups at higher risk of recurrence remains an important clinical issue. As stated in a communication from the subcommittee on control of anticoagulation of the Scientific and Standardization Committee of the International Society on Thrombosis and Haemostasis (ISTH), a risk of recurrence of appreciably higher than 5% at 1 year and 15% at 5 years would not be acceptable to many physicians and would usually discourage stopping OAT after VTE [7]. Accordingly, identifying risk factors of recurrent PE remains a major interest in order to improve risk stratification of these patients at intermediate risk of recurrence. This could enable the identification of subgroups with lower risk in whom indefinite OAT should be avoided, as well as higher-risk patients who may require indefinite OAT.

#### 1.1.2 Risk factors to predict VTE recurrence

Several clinical, biochemical, or imaging predictors of VTE recurrence have been identified, including age, male gender [8,9], obesity [10], some biologic thrombophilia [11,12], plasma D-dimer levels [13,14], or residual vein obstruction after deep vein thrombosis (DVT) [15]. However, the strength of the association with recurrent VTE was found to be moderate. Scores combining several risk factors have been proposed [[16], [17], [18], [19]], but they have been designed to determine the duration of treatment in patients with unprovoked PE, with an attempt to identify a subgroup of patients who would not require indefinite OAT. Predictors of recurrence in patients with provoked PE have been much less studied, and the challenge in this specific population would be to identify a subgroup of patients at higher risk who would benefit from long-term OAT.

#### 1.1.3 Residual pulmonary vascular obstruction to predict the risk of VTE recurrence

Residual pulmonary vascular obstruction (RPVO) can be defined as an incomplete repermeabilization of the pulmonary arteries after acute PE. RPVO is found in approximately 30% to 40% of patients after a first episode of PE [20]. Several studies have assessed RPVO as a predictor of VTE recurrence [19,[21], [22], [23], [24], [25]]. Most studies suggested that the presence of RPVO identified via lung ventilation/perfusion (V/Q) scintigraphy after a minimum of 3 months of OAT might be associated with a 2- to 3-fold increased risk of recurrent VTE. In a recent systematic review and meta-analysis of individual participant data in 809 patients with PE who underwent lung planar scintigraphy after at least 3 months of anticoagulation, the risk of recurrent VTE at 1 year was 5.8% (4.4%-7.2%) in patients with RPVO of <5%, compared with 11.7% (9.5%-13.8%) in participants with RPVO of ≥5% [26]. Of note is that 80.6% of patients had unprovoked PE.

However, some studies have methodologic limitations eg, inclusion of patients in long-term anticoagulation or unblinded use of V/Q scintigraphy for patients’ management. There is heterogeneity of studies in terms of population, VTE recurrence rate, or RPVO definition. The prognostic impact of RPVO was mostly reported in patients with unprovoked PE, for whom long-term anticoagulation is now recommended. There is limited data available on patients at an intermediate risk of recurrence. Finally, RPVO index was assessed in most of studies with V/Q planar scintigraphy while V/Q SPECT/CT imaging is currently widely used in daily practice.

#### 1.1.4 Why using V/Q SPECT/CT imaging to assess RPVO?

Most studies that assessed RPVO as a predictor of VTE recurrence used V/Q planar scintigraphy rather than computed tomography pulmonary angiography (CTPA) [27]. CTPA and V/Q scintigraphy rely on different approaches. CTPA provides morphologic information (partial or complete obstruction of a pulmonary artery branch), whereas V/Q scintigraphy shows functional consequences of PE on the regional pulmonary blood flow at an arteriolar level. In a meta-analysis published in 2019, RPVO as assessed with V/Q planar scintigraphy was associated with an increased risk of recurrent VTE. Although the analysis possibly lacked statistical power, RPVO was not predictive of recurrent VTE if it was assessed by CTPA [27]. Furthermore, CTPA has a higher radiation dose, especially to female breast, and requires the use of iodinated contrast media. Finally, the interpretation, comparison with previous scans, and quantification of RPVO are not simple with CTPA. In contrast, the interpretation of V/Q planar scintigraphy in the follow-up of PE does not require any particular expertise as it follows the same principles and semiological criteria as for the detection of acute PE, namely, the identification of mismatched perfusion defects.

In all studies that assessed RPVO as a predictor of VTE recurrence, RPVO was assessed with V/Q planar scintigraphy. However, a current limitation to the use of planar scintigraphy is high variability of RPVO definition in the literature. In some studies, lung planar scintigraphy was considered as abnormal if the RPVO was >5% [19], while in others, the cutoff was 10% [21] or 15% [22]. In a study including 65 participants for RPVO assessment with V/Q planar scintigraphy, κ coefficient for interobserver agreement between 2 nuclear medicine physicians was only 0.71 [24]. This might be explained by the inherent limitation of RPVO quantification using planar scintigraphy, which is only based on a visual estimation on 2-dimensional images.

Advances in imaging equipment and the development of radiotracers for ventilation have allowed the introduction of V/Q single photon emission computed tomography (V/Q SPECT/CT), a method of scintigraphic image acquisition that offers the advantage of 3-dimensional imaging, enabling more accurate quantification of RPVO [28]. V/Q SPECT/CT imaging may also open new perspectives for enabling automated quantification of RPVO. Importantly, V/Q SPECT/CT imaging, and not planar, is currently overwhelmingly used in daily practice in the follow-up of patients with PE [29].

### 1.2 Study aims

The primary aim of this study is to assess whether RPVO, evaluated with V/Q SPECT/CT imaging, is an independent predictor of recurrent VTE in patients with PE. This will be assessed in patients for whom anticoagulation is currently discontinued, but for whom there is a need to better predict the risk of recurrence in clinical practice. This includes patients with PE provoked by a minor transient risk factor and patients with unprovoked PE for whom anticoagulation need not be prolonged, regardless of why anticoagulation is not continued (benefit–risk balance, treating physician’s decision, and patient’s preferences). Given its technical advantages over planar imaging and because it is currently widely used in daily practice, V/Q SPECT/CT imaging will be used for RPVO assessment.

We also aim to assess the usefulness of performing a V/Q SPECT/CT scan after 3 to 6 months of OAT in various clinical situations. RPVO is very common after 3 to 6 months of OAT, and postembolic sequelae have the same pattern as acute PE on V/Q imaging. Thus, we aim to assess the diagnostic utility of a reference V/Q SPECT/CT scan performed after 3 to 6 months of anticoagulation in case of suspicion of PE recurrence. Finally, up to 50% of patients present with postpulmonary embolism syndrome, which is defined as new or progressive dyspnea, exercise intolerance, or impaired functional status after at least 3 months of anticoagulation following acute PE. We aim to study those patients with persistent dyspnea and impaired quality of life (QoL) after PE and to assess the role of V/Q SPECT/CT imaging in this setting.

## 2 Methods

### 2.1 Objectives

The primary objective is to assess whether RPVO, evaluated with V/Q SPECT/CT imaging after completion of 3 to 6 months of anticoagulation, is an independent predictor of recurrent VTE in patients with PE. The secondary objectives are listed in Table.

**Table:** Primary and secondary objectives of the PRONOSPECT cohort study.

Primary objective	
	To assess whether RPVO, evaluated with V/Q SPECT/CT imaging after completion of 3-6 mo of anticoagulation, is an independent predictor of recurrent VTE in patients with PE.
Secondary objectives	
VTE recurrence	To assess the cumulative risk of VTE recurrence 1 y and 2 y after discontinuation of anticoagulant therapy.
	To assess predictors for recurrent VTE other than RPVO, in order to be able to derive a predictive score.
	To assess the performances of scores already established to predict the risk of recurrence in patients with unprovoked PE, in patients at an intermediate risk of recurrence.
RPVO	To assess the proportion of patients with RPVO on V/Q SPECT/CT imaging after completion of 3 to 6 mo of anticoagulation.
	To assess different RPVO threshold on V/Q SPECT/CT imaging to predict the risk of VTE recurrence at 2 y.
	To assess predictors of RPVO at diagnosis.
	To compare technegas and krypton, the ventilation agents used in the study, for the assessment of RPVO after completion of 3 to 6 mo of anticoagulation.
Diagnosis of PE recurrence	To assess the safety of ruling out the diagnosis of PE recurrence with V/Q SPECT/CT imaging, using a baseline V/Q SPECT/CT scan performed after completion of 3 to 6 mo of anticoagulation.
Functional evaluation and quality of life	To assess the proportion of patients with dyspnea after completion of 3 to 6 mo of anticoagulation.
	To assess the proportion of patients with impaired QoL after completion of 3 to 6 mo of anticoagulation.
	To assess the association between RPVO and dyspnea or impaired QoL.
	To assess the proportion of patients with CTEPH.
	To assess whether RPVO, assessed with V/Q SPECT/CT imaging after completion of 3 to 6 mo of anticoagulation, is an independent risk factor for CTEPH.
	To assess the proportion of patients with chronic thromboembolic pulmonary disease.
	To assess the overall mortality of all causes during the 24-month follow-up.

CTEPH, chronic thromboembolic pulmonary hypertension; PE, pulmonary embolism; QoL, quality of life; RPVO, residual pulmonary vascular obstruction; V/Q SPECT, ventilation/perfusion single photon emission computed tomography; VTE, venous thromboembolism.

### 2.2 Study design

The PRONOSPECT trial is a prospective multicenter cohort study.

### 2.3 Participants

Inclusion criteria are as follows:

• Patients aged ≥18 years.
• Those who experienced an objectively proven PE.
• Those who have been treated initially with anticoagulant therapy for 3 to 6 uninterrupted months (180-210 days) and for whom anticoagulation will not be prolonged.

Noninclusion criteria are as follows:

• Unwilling or unable to give written informed consent.
• No social security affiliation.
• Isolated DVT.
• Other indication for long-term use of OAT (eg, atrial fibrillation and mechanic valve; preventing primary end-point assessment).
• Life expectancy < 6 months (preventing primary end-point assessment).
• Any patients for whom there is a high risk of recurrence and a strong indication to treat for longer than 6 months:
  - PE provoked by a major persistent factor, such as active malignancy or antiphospholipid antibody syndrome [30].
  - Recurrent unprovoked PE.
• PE provoked by a major transient risk factor [30] in patients for whom there is a very low risk of recurrence and a strong indication to propose short duration of OAT.

### 2.4 Procedure

Once a patient has consented to participate, a standardized baseline patient assessment will be performed, including the following:

• V/Q SPECT/CT imaging. Ventilation imaging will be performed using either ^81m^krypton gas or ^99m^Tc-technegas (Cyclomedica), an ultrafine particle that exhibits near-optimal distribution properties. These 2 ventilation agents allow for optimal ventilation SPECT acquisition [31,32]. V/Q SPECT/CT images should not be used for patients’ management.
• Collection of other potential predictor variables for VTE recurrence:
  - Demographic characteristics and risk factors for VTE.
  - Lab tests including D-dimer testing, drawn prior to discontinuation of OAT to mimic actual practice.
  - Lower limb compression ultrasonography if the patient had an associated DVT.
• Functional evaluation:
  - Dyspnea index using Modified Medical Research Council (MMRC) scale and New York Heart Association (NYHA) score.
  - Quality of life index using Pulmonary Embolism (PEmb)-Qol questionnaire.
  - Cardiac ultrasound in case of residual dyspnea or if the V/Q SPECT/CT demonstrates RPVO.

Patients will be included in the study the day of anticoagulation discontinuation and will be followed up in clinic or contacted by telephone every 6 months up to 2 years. Patients will be educated about the signs and symptoms of recurrent VTE and given emergency contact numbers and instructions if these signs were to arise in follow-up. In case of suspected PE recurrence, the diagnostic and therapeutic management will be left at the discretion of treating physician according to the availability of imaging modalities. When a V/Q SPECT/CT scan is performed, recurrent PE will be diagnosed is there is 1 segmental or 2 subsegmental new mismatched perfusion defects [33,34]. The study design is presented in Figure.

**Figure:**
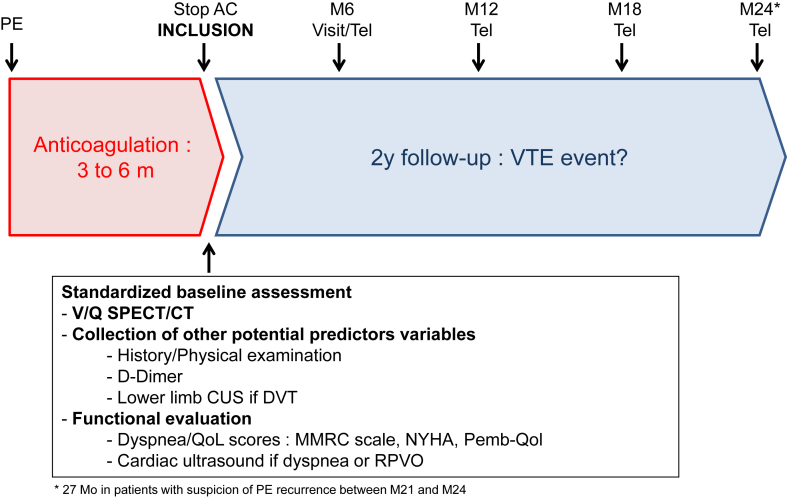
Study design. PE, pulmonary embolism; Pemb-QoL, pulmonary embolism quality of life; RPVO, residual pulmonary vascular obstruction; V/Q SPECT, ventilation/perfusion single photon emission computed tomography; VTE, venous thromboembolism.

### 2.5 Outcomes

The primary outcome is symptomatic recurrent VTE, including objectively confirmed nonfatal symptomatic PE or proximal DVT, or fatal PE during a 24-month follow-up after inclusion in the study [35]. All outcomes will be adjudicated blindly by an independent central Clinical Events Committee.

Secondary outcomes include following:

• Symptomatic recurrent VTE during a 3-month follow-up period in patients with suspicion of PE recurrence left untreated based on a negative V/Q SPECT/CT scan. All suspicion of PE recurrence will be adjudicated blindly by an independent central Clinical Events Committee.
• Dyspnea index assessed using MMRC scale.
• Quality of life assessed using Pulmonary Embolism (PEmb)-Qol questionnaire.
• Chronic thromboembolic pulmonary hypertension defined according to the European guidelines [36].
• CTEPD will be defined as follows: dyspnea/functional limitation after at least 3 months of OAT, RPVO on lung scintigraphy, no evidence of pulmonary hypertension (mPAM < 25) at rest, and no other conditions that may contribute to symptoms.
• Mortality of all causes.

### 2.6 Statistical analysis

#### 2.6.1 Variable analysis

Qualitative data will be presented as the number and the percentage of patients in each group (ie, patients with RPVO and patients with no RPVO). Quantitative data will be presented as mean and standard deviation, range, median, and IQR by group. Comparisons of qualitative variables between groups will be performed by a chi-square test or a Fisher’s exact test if required; comparison of quantitative data will be performed by the Student’s *t*-test or a nonparametric Wilcoxon test if required.

#### 2.6.2 Analysis of the primary outcomeTime to occurrence of recurrent VTE (primary outcome) is described in the 2 groups by Kaplan–Meier curves

The subject’s time-to-event will be censored at 2 years (end of the follow-up period), anticoagulation for another reason than recurrent VTE or loss to follow-up, whichever occurs first. Kaplan–Meier curves will be compared using a log-rank test and a Cox model will allow estimating adjusted *P* values and hazard ratios with 95% CIs, in order to take into account confounding factors. The assumption of proportional hazards over time in the Cox model will be verified using the cumulative sum of residuals method. The functional form of the association between continuous factors and the hazard of recurrence will be assessed using the fractional polynomials method. Missing data will be imputed using a multiple imputation method adapted for survival analysis. Sensitivity analyses will examine the association between imaging and the occurrence of recurrence using different cutoffs (10%, 15%, or 20%) and by maintaining the continuous measurement through both linear and nonlinear functional forms.

### 2.7 Sample size justification

The number of patients arises from comparison of the time to occurrence (censored by anticoagulation for another reason than recurrent VTE or loss to follow-up) of recurrent VTE, from the completion of anticoagulation. Based on the known recurrence risk of 4% to 7% at 1 year in patients at intermediate risk of recurrence and given that the study will also include patients with unprovoked PE whose recurrence risk is approximately 9% at 1 year, we estimated the recurrence risk in our population at 6% at 1 year (4% at 6 months and 8% at 2 years). Based on previous studies, we expect a VTE recurrence rate of 12% at 2 years in the group with RPVO (6% at 6 months) and 5% in the group without RPVO (2.5% at 6 months; hazard ratio, 2.5) [7,10,[31], [32], [33], [34]]. We anticipate that 10% of patients will be censored before 2 years (anticoagulation for another reason than recurrent VTE or loss to follow-up). We also expect that 40% of patients will have RPVO on V/Q SPECT/CT imaging, and 60% without RPVO. Thus, 52 events are required to ensure a power of 90%. Based on these assumptions, 655 patients need to be included.

### 2.8 Ethics and dissemination

The study has been approved by Ouest IV Ethics Committee (ID RCB: 2023-A01566-39) and registered in ClinicalTrial.gov registry (NCT06372730). Written informed consent will be obtained from all participants. The study commenced on June 2024.

### 2.9 Participant confidentiality

Any data obtained will be identified only by a participant identification number to maintain confidentiality. All records will be kept in a secured online database. If collected on paper, they will be kept in a locked cabinet. All data entry and manipulation will be performed by using participant identification numbers only.

## 3 Discussion/Limitations of the Study

The major interest of the study is the potential to demonstrate that RPVO is associated with an increased risk of VTE recurrence in patients at an intermediate risk of recurrence, which is currently unknown. V/Q SPECT/CT will not be used for patients’ management and the study will not include patients on OAT, allowing an independent evaluation of the prognostic significance of RPVO for VTE recurrence.

The study also has the potential to identify predictors of recurrence other than RPVO. This may allow the identification of subgroups of patients for whom the risk of recurrence would outweigh the risk of bleeding, who would benefit from lifelong anticoagulation.

We acknowledge that this study may have some limitations.

First, while there is currently no indication to prolong anticoagulation in patients with PE provoked by a minor transient risk factor, the decision to discontinue anticoagulation in some patients with unprovoked PE includes a significant subjective component and often involves various factors such as the estimated benefit–risk balance or the patient preference. This may have a direct influence on the study population’s inherent recurrence risk and homogeneity and limit the generalizability of findings. On the other hand, it is a pragmatic approach. The population corresponds to patients for whom there is a need to better predict the risk of recurrence. Similar population heterogeneity has been observed in other studies [37]. Furthermore, the population will be well documented at inclusion. Minor risk factors associated with PE and the reasons justifying the discontinuation of anticoagulant treatment in patients with unprovoked PE (patient preference, benefit–risk balance) will be collected. Thus, subgroup analyses could be conducted. Second, as patient’s management will not be based on RPVO or other potential predictors, this noninterventional study design will not allow us to assess the impact of modifying therapeutic management based on RPVO status. Furthermore, although performing a baseline V/Q scan after 6 months of anticoagulation could present various benefits, it remains a costly examination, with unequal accessibility, and is associated with exposure to radiation. One of the secondary objectives of the study is to assess predictors of RPVO. This may allow clinicians to target patients at increased risk of RPVO, who may benefit from a baseline V/Q lung scan, and, conversely, patients with a low risk of RPVO for whom the examination would not be justified. Finally, V/Q SPECT/CT scan performed at inclusion should remain available for interpretation of any suspicion of VTE recurrence. To limit a possible bias related to the knowledge of RPVO status, all suspicions of VTE recurrence (including the suspicion confirmed and ruled out) will be adjudicated blindly by an independent central Clinical Events Committee. Furthermore, to determine RPVO status, a central review of all V/Q SPECT/CT images will be performed independently by 2 nuclear medicine physicians who will be blinded to clinical history and the patient’s outcome. Any difference in interpretation will be resolved by consensus.
